# One-Step Microwave-Assisted Synthesis and Visible-Light Photocatalytic Activity Enhancement of BiOBr/RGO Nanocomposites for Degradation of Methylene Blue

**DOI:** 10.3390/ma14164577

**Published:** 2021-08-15

**Authors:** Kun-Yauh Shih, Yen-Ling Kuan, En-Rui Wang

**Affiliations:** Department of Applied Chemistry, National Pingtung University, Pingtung 90003, Taiwan; axw13671035@gmail.com (Y.-L.K.); transformersprime12345678@gmail.com (E.-R.W.)

**Keywords:** photocatalytic activity, bismuth oxybromide, graphene, microwave-assisted synthesis, nanocomposites

## Abstract

In this study, bismuth oxybromide/reduced graphene oxide (BiOBr/RGO), i.e. BiOBr-G nanocomposites, were synthesized using a one-step microwave-assisted method. The structure of the synthesized nanocomposites was characterized using Raman spectroscopy, X-ray diffractometry (XRD), photoluminescence (PL) emission spectroscopy, scanning electron microscopy (SEM), transmission electron microscopy (TEM), Fourier-transform infrared spectroscopy (FTIR), and ultraviolet-visible diffuse reflection spectroscopy (DRS). In addition, the ability of the nanocomposite to degrade methylene blue (MB) under visible light irradiation was investigated. The synthesized nanocomposite achieved an MB degradation rate of above 96% within 75 min of continuous visible light irradiation. In addition, the synthesized BiOBr-G nanocomposite exhibited significantly enhanced photocatalytic activity for the degradation of MB. Furthermore, the results revealed that the separation of the photogenerated electron–hole pairs in the BiOBr-G nanocomposite enhanced the ability of the nanocomposite to absorb visible light, thus improving the photocatalytic properties of the nanocomposites. Lastly, the MB photo-degradation mechanism of BiOBr-G was investigated, and the results revealed that the BiOBr-G nanocomposites exhibited good photocatalytic activity.

## 1. Introduction

Since 1972, the principle and applications of photocatalysis have been extensively investigated. Recently, with the increase in environmental protection awareness, photocatalysis has attracted significant attention for the treatment of environmental pollution and the conversion of solar energy [1,2,3]. As an advanced oxidation process, photocatalysis generates electron–hole pairs via light irradiation of photocatalysts. This photocatalytic degradation reaction can be attributed to the excitation of electron radiation in the valence band (VB) to the conduction band (CB) and the formation of holes with positive charges in the VB. During photocatalysis, the adsorbed material further reacts with the electron and hole to produce numerous oxidized substances that can degrade some organic pollutants [4,5].

Titanium dioxide (TiO_2_) is a traditional photocatalyst with high photocatalytic activities and low toxicity [6,7]. Currently, TiO_2_ is the most commonly used photocatalytic material owing to its low toxicity, which significantly limits its negative impact on the environment [8]. However, the large band gap of TiO_2_ (3.2 eV) limits its ability to effectively convert solar energy. This is because it can only be excited by ultraviolet (UV) light (λ < 390 nm), which makes up only approximately 7% of the solar spectrum [9]. To increase the effective utilization of solar energy, researchers have devoted tremendous efforts to developing a new photocatalyst that can perform photocatalysis in the visible light range and clean the environment in an effective and environmentally friendly manner [10]. Bismuth oxyhalides (BiOX, X = Cl, Br, and I) have attracted increased attention for the visible light photocatalytic degradation of organic pollutants [11,12]. Liu et al. [13] combined BiOI with BiOCl or BiOBr to prepare BiOI/BiOCl, which exhibited a methyl orange (MO) removal of 78% after 150 min visible light irradiation. In addition, Meng et al. [14] utilized Pd surface-modified BiOBr nanoparticles to degrade phenol and found that the nanoparticles exhibited a 100% phenol removal after 300 min visible light irradiation. However, the recombination of the photogenerated electrons and holes of BiOX reduces the photocatalytic activity of the catalyst [15]. The high electron and hole recombination rates of BiOX have restricted its further application. [16,17].

Graphene is a well-known special material globally [18]. The lifetime of the electron–hole pairs of a photocatalyst can be enhanced by adding graphene materials to the photocatalyst. This is because the high conductivity of graphene reduces charge recombination [19]. Consequently, graphene-based photocatalysts have attracted tremendous attention owing to their photocatalytic efficiency. For example, Liang et al. [20] synthesized NiFe_2_O_4_-reduced graphene oxide (RGO) nanocomposites and found that these materials exhibited 99.1% methylene blue (MB) removal under 180 min UV irradiation. In addition, Liu et al. [21] prepared an RGO-wrapped TiO_2_ hybrid and found that the catalyst exhibited 100% MB removal under 150 min UV irradiation. Furthermore, Patil et al. [22] synthesized BiVO_4_/Ag/rGO hybrid architectures and found that the catalyst exhibited approximately 90% MB dye removal.

Several methods, such as ultrasonication, hydrothermal method, solvent heat method, and the sol-gel method, are used for the synthesis of photocatalysts. In a previous study, an H_3_PW_12_O_40_/TiO_2_ composite photocatalyst was prepared using a high-intensity ultrasonication method at a low temperature (80 °C). The H_3_PW_12_O_40_/TiO_2_ photocatalyst exhibited a 95% MB degradation rate under 90 min solar irradiation [23]. In addition, Vadivel et al. [24] synthesized an Sm-BiOBr/rGO composite photocatalyst using a solvothermal method by utilizing methanol as the solvent. The synthesized photocatalyst exhibited promising potential for the degradation of various hazardous chemicals and organic pollutants. Behera et al. [25] synthesized a series of ZnFe_2_O_4_@RGO nanocomposites using hydrothermal and calcination methods and investigated their applications for the degradation of ciprofloxacin. Farhadian et al. [26] synthesized N, S-doped TiO_2_ (NST), N, S-doped ZnO (NSZ), and their composites with chitosan (NST/CS, NSZ/CS) using the sol gel-hydrothermal method. They found that NST/CS exhibited the highest tetracycline degradation efficiency of 91% under 20 min visible light exposure. Chamjangali et al. [27] prepared nanoflower-like Ag-ZnO photocatalysts using a photoreduction and solution precipitation method and investigated their application for the photocatalytic degradation of MO and MB. Kumar et al. [28] synthesized Ag/TiO_2_ by dispersing Ag nanoparticles into ethanol under sonication, after which TiO_2_ was added to the ethanol solution. The synthesized catalyst exhibited the photocatalytic degradation of MB under UV-C light irradiation. Liang et al. [29] synthesized magnetic Fe_3_O_4_@BiOI@AgI spheres using a multi-step process. The synthesized spheres exhibited excellent visible light driving activity against RhB, BPA, and *E. coli* cells. Sanaa et al. [30] synthesized yBiOBr-(1−y)BHO heterojunction using hydrothermal synthesis and solution mixing methods, and the heterojunction exhibited enhanced visible-light photocatalytic properties. Some researchers have synthesized nanocomposites by combining BiOBr and graphene and investigated their potential for the degradation of organic pollutants in wastewater. Jiang et al. [31] synthesized BiOBr-RGO nanocomposites using the hydrothermal method, and they found that the nanocomposites exhibited 100% nitrobenzene degradation after 360 min visible light irradiation. These studies indicate that the introduction of RGO to BiOBr could enhance its visible-light photocatalytic activity. Janani et al. [32] synthesized a magnetic RGO–BiOBr (MRGO–BiOBr) composite by subjecting MRGO and BiOBr to ultrasonication separately for 30 min, after which they were mixed together under magnetic stirring for 24 h. The effects of parameters, such as catalytic dose and initial dye concentration, were investigated under visible light irradiation. The results revealed that the composite exhibited 76.24% MB dye removal after 120 min visible light irradiation.

However, the large energy consumption and long reaction time of these methods have limited their further application. To overcome these challenges, in this study, we employed a simple, efficient, time-saving, and environmentally friendly, one-step synthesis method for the synthesis of photocatalyst nanocomposites. The fabrication of nanomaterials using microwaves is an environmentally friendly and cost-effective method. In addition, the microwave-assisted method can obtain high-purity products, improve product yields, and increase reproducibility [33]. In this study, BiOBr, a photocatalyst with visible light photocatalytic ability, was selected and combined with RGO to synthesize a BiOBr/RGO (BiOBr-G) photocatalyst. Furthermore, the microwave-assisted method was employed to synthesize BiOBr-G within short durations, and the photocatalytic activity of these nanomaterials was investigated. In addition, the crystal structure, morphology, functional groups, absorption spectra, and photocatalytic mechanism were investigated using various methods. The photocatalytic activity of the nanocomposites was investigated by evaluating their MB degradation ability. BiOBr and BiOBr-G photocatalysts were successfully synthesized, and their pollutant degradation abilities were investigated. Lastly, their effects on electron–hole pair separation were also discussed.

## 2. Materials and Methods

### 2.1. The Materials Used

Bismuth nitrate Bi(NO_3_)_3_·5H_2_O was obtained from Sigma-Aldrich (St. Louis, MO, USA). Potassium bromide (KBr) and natural graphite powder (99.99%, metals basis) were purchased from Alfa Aesar (Haverhill, MA, USA). All of the chemicals used in the study were of analytical grade. The aqueous solutions were prepared using deionized (DI) water.

### 2.2. Preparation of Graphene Oxide 

In this study, GO was synthesized from graphite powder using the modified Hummers method [34]. Briefly, 70 mL of concentrated sulfuric acid (Japan Chemicsl Industries Co. Ltd, Shimizu-ku, Japan ) was placed in an ice bath and cooled to 5 °C. Subsequently, graphite powder, NaNO_3_, and KMnO_4_ were added into the flask and stirred evenly for 2 h. Thereafter, 300 mL of DI water and 10 mL of 30% hydrogen peroxide were added to the solution to stop the reaction. Subsequently, the solution was subjected to suction filtration, after which the product was placed in 500 mL of 5% hydrochloric acid and stirred 30 min to remove the remaining metal ions. Thereafter, the HCl solution was removed and washed with DI water several times until the pH of the GO suspension was neutral. Lastly, the suspension was filtrated and dried in an oven for 12 h at 70 °C to obtain the GO powders.

### 2.3. Synthesis of the BiOBr-G Nanocomposites

**Figure 1:**
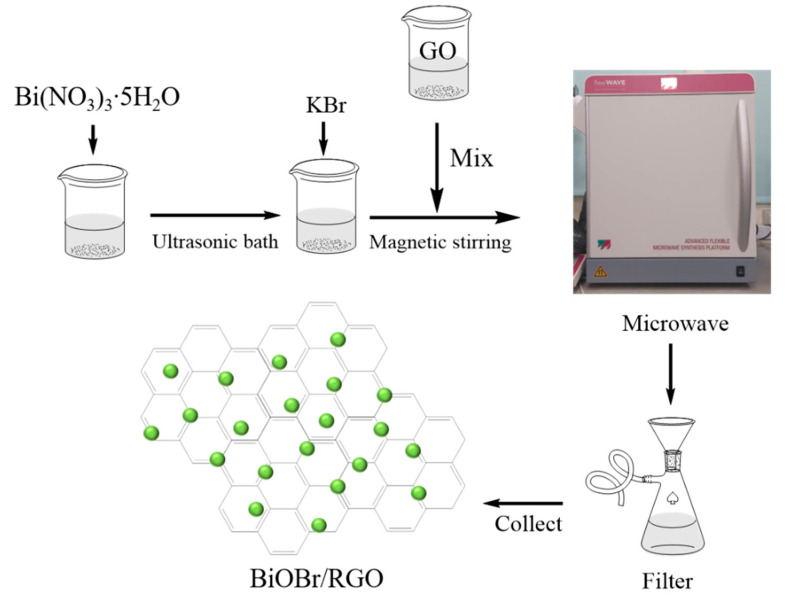
Schematic illustration of the synthesis process of the BiOBr-G nanomaterial.

BiOBr-G nanocomposites with various GO weight percentages were synthesized using the microwave-assisted method. Briefly, 0.4608 g Bi(NO_3_)_3_·5H_2_O was dissolved in 25 mL of ethylene glycol, after which the mixture was dispersed in an ultrasonic bath for 15 min. Simultaneously, GO powders were dissolved in 10 ml of ethylene glycol, after which the mixture was subjected to magnetic stirring for 30 min. Subsequently, 0.2380 g of KBr was added to the Bi(NO_3_)_3_·5H_2_O solution, after which the mixture was stirred at room temperature for 0.5 h. Thereafter, the GO solution was carefully added to the afore-mentioned solution, and the solution was stirred continuously for 30 min. Subsequently, the mixture was transferred into a 50 mL Teflon-lined vessel, and the mixture was maintained at 90 °C for 15 min. The precipitates were collected by centrifugation and washed three times with DI water and ethanol. Thereafter, the precipitates were dried in an oven at 80 °C overnight. For comparison, pure BiOBr and RGO were also prepared using a similar process. The BiOBr-G nanocomposites with RGO content of 0.5, 1, and 5 wt% were labeled as BiOBr-G0.5, BiOBr-G1, and BiOBr-G5, respectively. The schematic illustration of the synthesis process is shown in Figure 1.

### 2.4. Photocatalytic Activity

The photocatalytic efficiency of the BiOBr-Gs nanocomposites was investigated. The photocatalytic reactions of the nanocomposites were investigated using a PCX50B Discover multi-channel photoreactor (Perfect Light Technology Ltd, Beijing, China). A 5 W white light Light-emitting diode lamp was used as the light source to provide visible light with a wavelength of above 420 nm. The MB used in this study was a model pollutant that must be removed. To prepare the sample used for the photocatalytic analysis, 30 mg of as-prepared samples was weighed into a quartz flask, after which 50 mL of 2 × 10^−5^ M MB solution was added. Subsequently, the solution was stirred in the dark for 10 min to achieve adsorption–desorption equilibrium, after which the solution was subjected to irradiation. The photocatalytic reaction was carried out under visible light irradiation for 75 min, and 3 mL of the as-prepared solution was collected every 15 min. The collected MB solutions were centrifuged (14,000 RPM, 3 min) to remove the sample powder. The degradation of MB was evaluated by measuring the characteristic absorption of MB solution at 664 nm using a CT-2200 UV-vis spectrophotometer (ChromTech Co. Ltd; Apple Valley, MN, USA). After measurement, the collected samples were re-injected to the quartz flask, and the experimental condition was maintained.

The removal efficiency of the target pollutant was determined using the following equation:

(1) $$\mathrm{Removal}\;\left(\%\right)=\left(1-\frac{C_{t}}{C_{0}}\right)\times 100\%$$

where C_0_ and C_t_ are the initial concentration and concentration of MB at time t, respectively.

### 2.5. Characterization

The as-synthesized BiOBr-G nanocomposites were synthesized using a microwave (Flexiwave T660, Milestone srl, Sorisole, Italy). The crystal structure of the as-synthesized nanocomposites was analyzed using powder X-ray diffraction (XRD, D8A25 eco, BRUKER Co. Ltd, Billerica, MA, USA) with CuKα X-ray radiation (λ = 1.5418 Å) operated at 40 kV and 25 mA. The morphology of the particles was observed using transmission electron microscopy (TEM, Hitachi H-7500, Tokyo, Japan) at an accelerating voltage of 80 kV. The surface morphology of the samples was investigated using scanning electron microscopy (SEM, Jeolism -6930, Tokyo, Japan) equipped with a system of energy-dispersive spectroscopy (EDS, INCAx act, Munich, Germany). The photoluminescence (PL, Hitachi F-7000, Tokyo, Japan) spectra of the samples were obtained using a Hitachi F-7000 (Tokyo, Japan) spectrometer at an emission wavelength of 300 nm. The Raman spectra were determined using a Princeton Instruments Acton SP2500 (Acton, MA, USA) monochromatic/photographic spectrometer equipped with a nitrogen-cooled CCD detector and a Spec-10 system. The light absorption properties of the samples were investigated using UV−vis diffuse reflectance spectroscopy (DRS, JASCO IBXL0005-V770-EA, JASCO, Tokyo, Japan). The chemical state of the composites was measured using Fourier-transform infrared spectrometer (FT-IR, JASCO FT/IR-6700, JASCO, Tokyo, Japan). A multi-channel photochemical reaction system (Perfect Light Technology Ltd, PCX-50B, Beijing, China) was used during the photocatalytic reaction experiment. The MB concentration was measured using a CT-2200 ultraviolet-visible (UV-Vis) spectrophotometer (ChromTech Co. Ltd.; Apple Valley, MN, USA). 

## 3. Results

### 3.1. Characterization of BiOBr and BiOBr-G

#### 3.1.1. XRD Analysis

**Figure 2:**
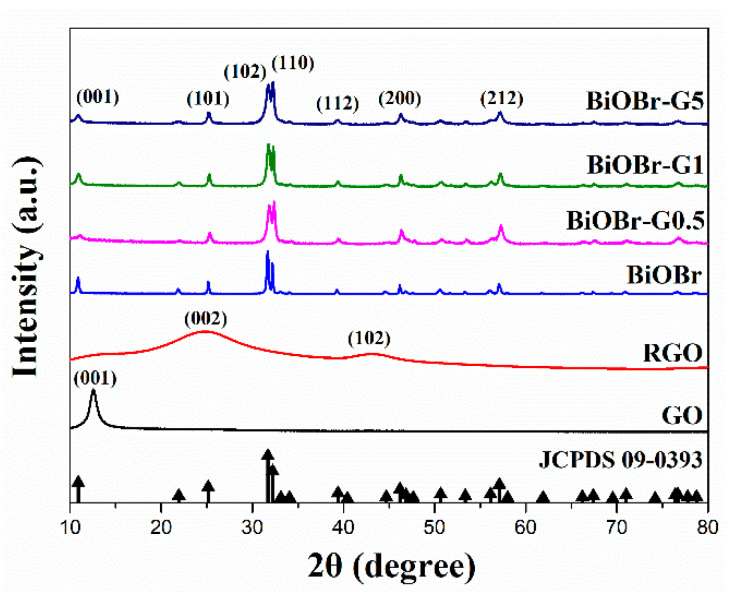
The XRD spectra of GO, RGO, BiOBr, and BiOBr-G samples (Black arrow show a standard card JCPDS 09-0393 of pure BiOBr).

The GO, RGO, BiOBr, and the BiOBr-G nanocomposites synthesized using the microwave-assisted method were analyzed using XRD (Figure 2). The XRD pattern of the GO exhibited a distinct strong reflex at 2θ = 12.6°, corresponding to the (001) crystal plane of GO. In addition, notable peaks were observed in the XRD pattern of the RGO at 2θ = 25.0°and 43.1°, corresponding to the RGO (002) and (102) crystal plane, respectively [35]. However, the (001) crystal plane of GO was not observed in the XRD pattern of the RGO, confirming the successful reduction of GO by the microwave-assisted method. The XRD patterns of BiOBr and BiOBr-G nanocomposites were consistent with the standard card number of pure tetragonal phase BiOBr (JCPDS 09-0393) [36]. The main peaks of the XRD pattern were observed at 2θ = 10.9°, 25.3°, 31.7°, 32.3°, 39.3°, 46.3°, and 57.2°, corresponding to the (001), (101), (102), (110), (112), (200), and (212) crystal planes, respectively [37]. However, the (001) crystal plane of GO was not observed in the XRD patterns of all the BiOBr-G samples, confirming the successful reduction of GO. Furthermore, the typical diffraction peaks of RGO at 2θ = 25.0° and 43.1° were not observed in the XRD patterns of the BiOBr-G nanocomposites, which could be attributed to the relatively low diffraction peak intensity of RGO [38].

#### 3.1.2. Raman Spectrum

**Figure 3:**
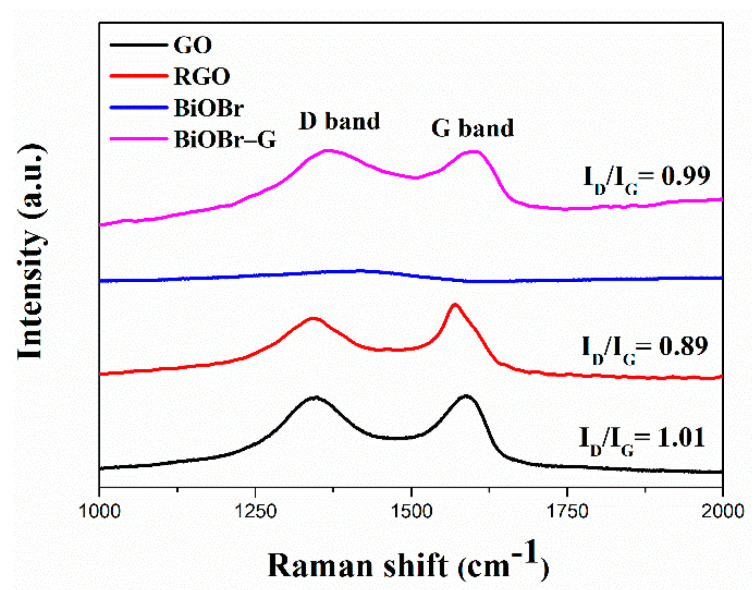
Raman spectra of the GO, RGO, BiOBr, and BiOBr-G nanocomposites. I_D_ / I_G_ is the ratio of the integrated intensities of the D and G bands.

Figure 3 shows the Raman spectrum of GO, RGO, and BiOBr-G. Two notable bands, the D and G bands, were observed in the Raman spectra of GO and RGO, respectively. The D band corresponded to the k-point phonon mode, which could be attributed to the sp^3^ defects in the carbon material, such as vacancies and edge effect. The G band could be attributed to the sp^2^ carbon atoms vibration model [39,40]. The D and G bands of GO were observed at 1348 cm^−1^ and 1587 cm^−1^, respectively. In addition, the D and G bands of RGO were observed at 1343 cm^−1^ and 1572 cm^−1^, respectively. However, no notable peak was observed in the Raman spectrum of BiOBr. In addition, two bands were observed in the Raman spectrum of BiOBr-G at 1365 and 1601 cm^−1^. The D band to G band intensity ratio of BiOBr-G (I_D_/I_G_ = 0.99) was slightly lower than that of the pure GO (I_D_/I_G_ = 1.01). This confirms the decrease in the sp^3^ domain of the carbon atoms in BiOBr-G and the increase in the production of the graphene sp^2^ structure in BiOBr-G. According to the literature, these results correspond to the high electron transport rate of BiOBr-G [41,42]. In addition, the I_D_/I_G_ value of BiOBr-G was higher than that of RGO (I_D_/I_G_ = 0.89), indicating that the RGO loaded with BiOBr has more defects than RGO. The shift of the D and G band in the BiOBr-G Raman spectrum confirmed that the microwave-assisted synthesis achieved both the reduction of GO and the formation of Bi-OBr-G [24,43].

#### 3.1.3. FTIR Spectroscopy

**Figure 4:**
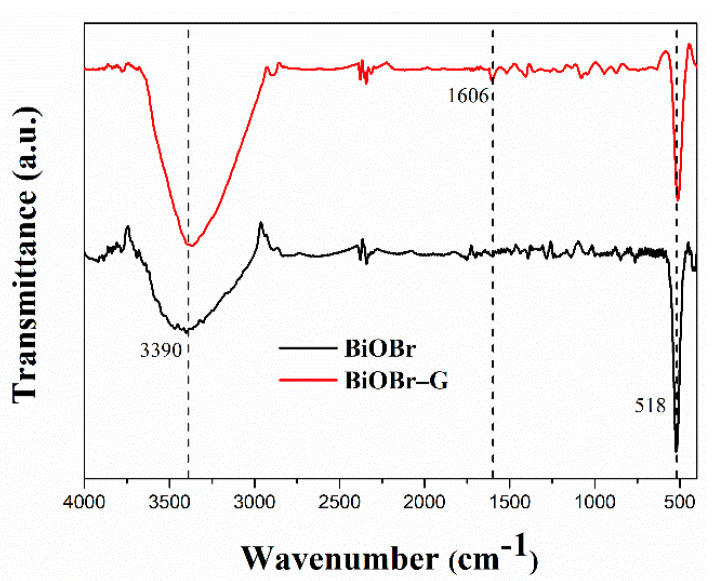
The FTIR spectrum of BiOBr and BiOBr-G nanocomposites.

The FT-IR spectra of BiOBr and BiOBr-G nanocomposites are shown in Figure 4. A notable band was observed in the FT-IR spectra of the BiOBr nanosheets at 514 cm^−1^, which could be attributed to the typical symmetric A_2u_ type of the Bi–O bond vibrations. In addition, a similar band was observed in the FT-IR spectra of the BiOBr-G nanocomposite [44,45]. Furthermore, a wider absorption band was observed in the FT-IR spectra of these samples at 3404 cm^−1^, which could be attributed to the O–H stretching mode of adsorbed water or hydroxyl groups [46,47]. Moreover, the BiOBr-G sample exhibited the typical FT-IR spectrum of BiOBr-G. This indicates that the addition of GO to BiOBr during the microwave-assisted synthesis had no effect on the crystal structure of BiOBr [48]. 

#### 3.1.4. Morphological Characterization

**Figure 5:**
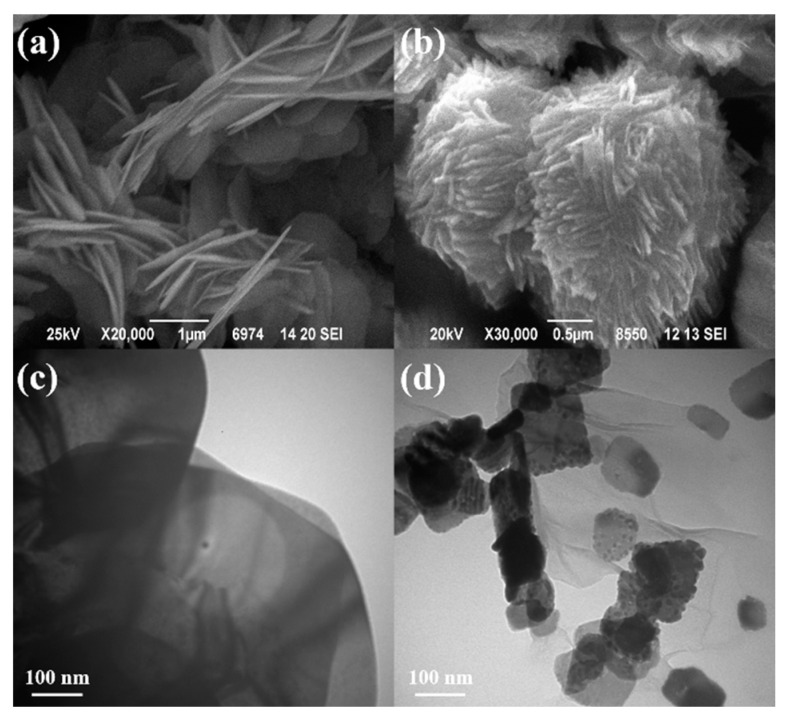
SEM images of (**a**) BiOBr and (**b**) BiOBr-G. TEM images of (**c**) BiOBr and (**d**) BiOBr-G.

Figure 5a,b show the SEM image of BiOBr and BiOBr-G nanocomposites. The pure BiOBr exhibited a thin flake structure with a two-dimensional relatively smooth surface, which was assembled to flower-like microstructures [49]. However, the BiOBr-G nanocomposite exhibited a spherical structure formed by the assembly of smaller and denser nanosheets. This indicates that the addition of RGO affected the crystallization process of BiOBr and destroyed the existing micro-flower nanostructure of BiOBr, thus increasing the dispersion and photocatalytic activity of BiOBr-G [50]. Furthermore, TEM was conducted to investigate the structural characteristics of the BiOBr and BiOBr-G nanocomposites, and the results are shown in Figure 5c, d. As shown in Figure 5c, BiOBr exhibits a thin sheet stacked structure with a flat surface, which is consistent with the SEM results and observation [51]. After the addition of RGO, the particle size of BiOBr reduced significantly, and the dispersion on the wrinkled RGO increased. This indicated the successful preparation of BiOBr-G. This is because RGO effectively controlled the crystal size of BiOBr and prevented the agglomeration of nanoparticles [52]. In addition, after the addition of RGO, the surface area of the BiOBr-G nanocomposite in contact with dye increased compared to that of pure BiOBr owing to its smaller particle size. Consequently, the electron transfer and photocatalytic activity of BiOBr were significantly enhanced [53].

#### 3.1.5. UV-Vis Diffuse Reflectance Spectra

**Figure 6:**
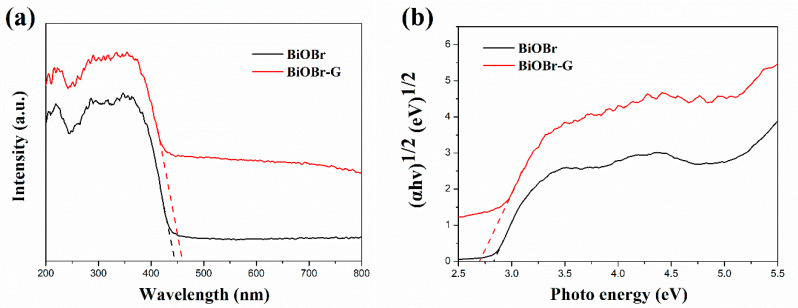
(**a**) Diffuse reflectance spectra (DRS) and (**b**) Tauc plots of the BiOBr and BiOBr-G nanocomposites.

Figure 6a shows the UV-Vis diffuse reflectance spectra (DRS) results of BiOBr and BiOBr-G nanocomposites. As shown in the image, both BiOBr and BiOBr-G exhibit a wide and high absorption in the visible light range. In addition, the absorption peak of BiOBr was observed at 445 nm. After adding RGO, the BiOBr-G absorption edge slightly red-shifted to 462 nm. The optical band gap (E_g_) of the BiOBr and BiOBr-G composites was obtained using the Tauc relation, as follows [54]:

αhν = A(hν − E_g_)^n/2^ (2)

where A is a constant, which depends on the transition probability; h is the Planck constant; ν is the frequency of light; α is the absorption coefficient; and n is based on the transition property of the semiconductor [55]. For example, n = 2 corresponds to the indirect transition of the semiconductor, whereas n = 1/2 corresponds to the direct transition of the semiconductor. Previous studies have reported that the n of BiOBr is 2, indicating that the transition property of BiOBr is indirect [56]. The plot of (αhν)^1/2^ vs. the photon energy is shown in Fig. 6b. As shown in the image, the E_g_ value of the BiOBr and BiOBr-G nanocomposites are 2.83 and 2.69 eV, respectively. The E_g_ value of the BiOBr is similar to the previous study [24].

#### 3.1.6. PL Analysis

**Figure 7:**
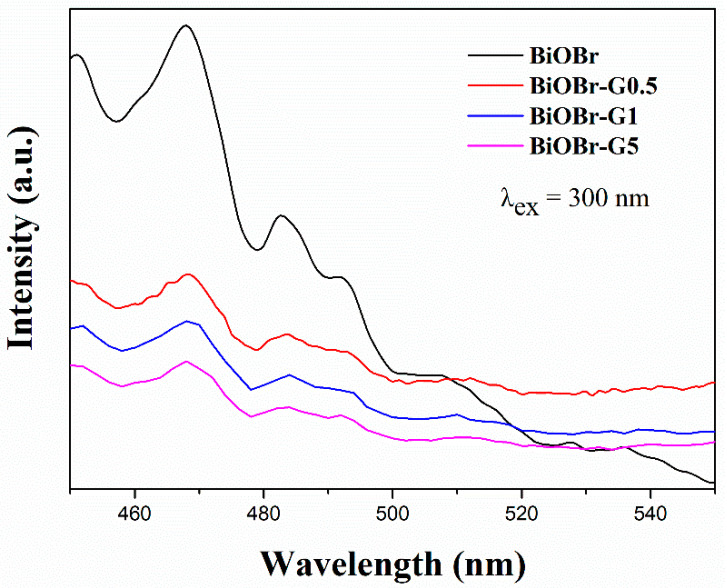
PL spectra of the as-synthesized samples. λ_ex_ is the wavelength of fluorescence excitation.

The PL spectra of BiOBr and BiOBr-G were obtained to investigate their electron–hole recombination properties. With an increase in the PL intensity of a sample, its photon separation rate decreases, thus increasing the electron–hole pair recombination. Consequently, this reduces the photocatalytic activity of the sample [57]. Figure 7 shows the PL spectrum of the as-synthesized BiOBr and BiOBr-G nanocomposites at an exciting wavelength (λ_ex_) of 300 nm. The highest peaks of BiOBr and BiOBr-G were observed at 468 nm. However, the PL spectral intensity of the pure BiOBr was significantly stronger than that of BiOBr-G. With an increasing in the RGO content, the intensity of the BiOBr-G emission peak decreased, indicating that the addition of RGO enhanced the separation of carriers. These results confirmed that photoelectrons moved from BiOBr to RGO and that RGO facilitated the suppression of the electron–hole pair recombination [58]. This indicates that a higher quantum efficiency can be achieved during photocatalytic reactions by utilizing the hierarchical structure of BiOBr-G.

### 3.2. Photocatalytic Activity

**Figure 8:**
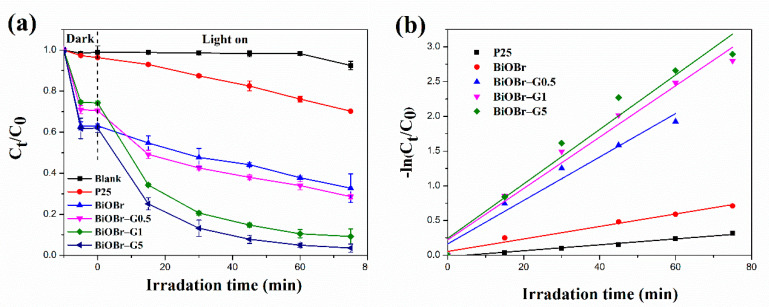
(**a**) Photocatalytic degradation of MB by P25, BiOBr, and BiOBr-Gs. (**b**) Photocatalytic kinetic of P25, BiOBr, and BiOBr-Gs.

To investigate the photocatalytic properties of the fabricated samples, the MB degradation properties of commercial TiO_2_ (P25), BiOBr, and each BiOBr-G sample under visible light were investigated (Figure 8a). The results revealed that the pure MB did not exhibit self-degrading properties under visible light; however, the addition of the photocatalyst significantly improved the removal efficiency [59]. The order of MB removal percentage of each material was: TiO_2_ P25 (29.74%) < BiOBr (67.25%) < BiOBr-G0.5 (71.37%) < BiOBr-G1 (90.80%) < BiOBr-G5 (96.41%). The photocatalytic activity of P25 under visible light was lower than those of the other samples, which could be attributed to the fact that the band gap of P25 is higher than that of BiOBr and BiOBr-Gs. This indicates that the P25 sample exhibited the lowest MB removal rate compared to the other samples. In addition, the photocatalytic activity of all the BiOBr-G nanocomposites was significantly higher than that of BiOBr. These findings are consistent with the PL spectra in Figure 7. Furthermore, the photoelectron–hole pair separation efficiency of the BiOBr-G sample was higher than that of the BiOBr sample, and it also exhibited an optimum photocatalytic activity performance. The addition of RGO to BiOBr significantly enhanced the photocatalytic activity of BiOBr, with the optimum RGO concentration being 5 wt%. In addition, the photo-induced electron transfer rate from the BiOBr surface to the RGO surface increased with an increase in the RGO loading, thus increasing the photocatalytic activity of the BiOBr-G nanocomposite. The prepared photocatalyst follows the first-order kinetic model (Figure 8b), which can be expressed using the following equation [60]:

(3) $$-\ln\frac{C_{0}}{C_{t}}={\mathrm{kt}}^{\;}$$

where k and t are the rate constant and lighting time, respectively. The k values of P25, BiOBr, BiOBr-G0.5, BiOBr-G1, and BiOBr-G5 are 0.00426, 0.0090, 0.0313, 0.0370, and 0.0392 min^−1^, respectively. The increase in the k value of BiOBr-G compared to that of BiOBr indicates the short-term degradation of MB dye. In addition, the BiOBr-G5 nanocomposite exhibited the optimum photocatalytic effect, which was 4.36 times higher than that of BiOBr. This result indicates that the addition of graphene to BiOBr significantly improved the photocatalytic efficiency of BiOBr [61]. Table 1 shows the comparison of the MB removal and rate constant of BiOBr-G5 to those of different materials investigated in previous studies. The MB-removal percentage of BiOBr-G5 was similar to that of other materials; however, the rate constant of BiOBr-G5 was higher than those of the other materials. This result illustrates the enhanced MB-removal efficiency of BiOBr-G5.

### 3.3. Photocatalytic Mechanisms

**Figure 9:**
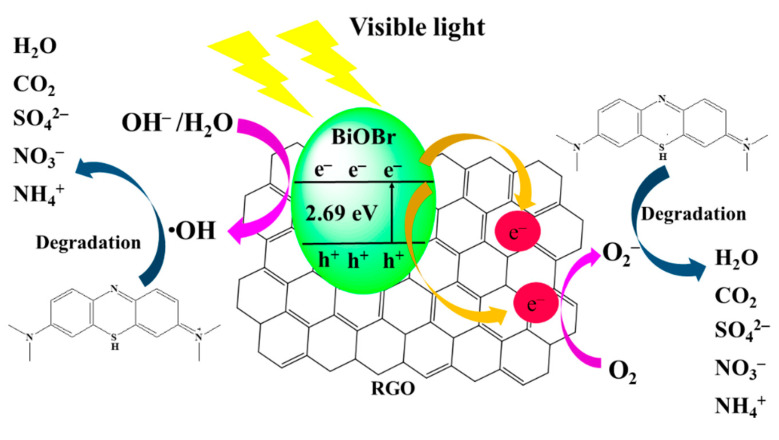
The photocatalytic mechanism diagram.

Figure 9 shows the photocatalysis mechanism of BiOBr-G under visible light irradiation. The excitation of the electrons (e^−^) of the VB into the CB under visible light irradiation leads to the generation of electron–hole pairs. The E_CB_ and E_VB_ of BiOBr were evaluated using the equation below [67]:

E_VB_ = χ − E_e_ + 0.5E_g_ (4)

E_CB_ = E_V_ − E_g_ (5)

According to the DRS measurements, E_e_ is 4.5 eV, and it represents the energy of free electrons on the hydrogen scale. In addition, E_VB_ and E_CB_ correspond to the edge potentials of VB and CB, respectively. From the DRS results, χ is semiconductor electronegativity. The χ of BiOBr was 6.17 eV, and the E_g_ of BiOBr-G was 2.69 eV. Using the afore-mentioned equation, the E_VB_ and E_CB_ of BiOBr are approximately 3.02 and 0.33 eV, respectively [68]. The VB position of BiOBr is above OH^−^/•OH (2.38 eV to Normal Hydrogen Electrode, NHE) and H_2_O/•OH (2.72 eV to NHE) [69]. The photogenerated electron-hole of BiOBr can oxidize OH^−^ and H_2_O to the •OH free radicals, and •OH can degrade the MB molecules.

The RGO exhibits an extremely efficient electron collection and separation structure. After the charge separation of BiOBr, the excited electrons were transferred to the RGO surface, thus decreasing the rate of electron–hole recombination [70]. Photogenerated electrons reacted with O_2_ molecules adsorbed on the surface of the RGO to produce O_2_^−^ radicals. Simultaneously, the OH^−^ and H_2_O reacted with the holes in the VB, thus generating •OH radicals. The RGO nanosheets enhanced the electron–hole pair separation and also increased the adsorption of reactants owing to π–π interactions and electrostatic force effects. In addition, the high surface area of RGO facilitated the adsorption of MB and the active oxides (O_2_^−^ and •OH radicals), thus enhancing the contact probability with radical and dye molecules and enhancing the photocatalytic activity of the catalyst. Finally, the MB molecules were degraded by O_2_^−^ and •OH radicals, thus producing non-toxic small molecules (H_2_O, CO_2_, SO_4_^2−^, NO_3_^−^, and NH_4_^+^). This photocatalytic process can be represented using the following chemical equation [20,71].

BiOBr + hν → e− (BiOBr) + h^+^ (BiOBr) (6)

e^−^ + O_2_ → O_2_^−^ (7)

h^+^+ OH^−^/H_2_O → •OH (8)

h^+^/ O_2_^−^/ •OH + C_16_H_18_N_3_ClS → H_2_O + CO_2_ + SO_4_^2−^ + NO_3_^−^ + NH_4_^+^ (9)

## 4. Conclusions

In summary, in this study, BiOBr-G nanocomposites were successfully synthesized using a facile one-step microwave-assisted method and characterized by XRD, Raman, FTIR, SEM, TEM, UV-vis DRS, and PL. The XRD analysis confirmed the successful synthesis of GO, RGO, BiOBr, and all the BiOBr-G nanocomposites. However, the RGO diffraction peak was not observed in the XRD pattern of BiOBr-G. In addition, D and G bands were observed in the Raman spectrum of the BiOBr-G nanocomposites, indicating the graphene structure of the synthesized nanocomposite. Furthermore, a Bi–O vibrational peak was observed in the FT-IR spectra of BiOBr and Bi-OBr-G. The SEM and TEM results revealed that the presence of RGO in the BiOBr-G nanocomposites prevented the nanoparticles’ agglomeration and reduced the particle size. In addition, the band gap of BiOBr-G was lower than that of BiOBr, indicating that the addition of RGO to BiOBr enhanced the photocatalytic activity of BiOBr. The PL results revealed that BiOBr-G exhibited higher quantum efficiency during the photocatalytic process compared to BiOBr. The photocatalytic activity of the as-synthesized nanocomposites under visible light irradiation was investigated. BiOBr-G5 exhibited the optimum photocatalytic activity, and the removal percentage of MB achieved more than 96% in 75 min. The prepared photocatalyst follows the first-order kinetic model, and the rate constant of BiOBr-G5 (0.0392 min^−1^) was higher than those of the other materials. These results were also superior to those of various catalysts reported in previous studies. The BiOBr-G nanocomposite synthesized in this study is an efficient, cost-effective, and environmentally friendly photocatalyst, with promising potential for wastewater treatment. 

## Figures and Tables

**Table 1:** MB removal rate and kinetic constant of various catalysts reported in previous studies.

Atalyst	MB Removal (%)	Rate Constant (min^−1^)	References
BiOBr-G5	96.41	0.0392	This work
NiFe_2_O_4_-RGO	99.1	0.0199	[20]
Ag-ZnO	~100	—	[27]
Ag/TiO_2_	36~90	0.001~0.008	[28]
BG-6	100	0.0087	[31]
NiFe_0.5_Nd_1.5_O_4_	93.4	—	[62]
MnFe_2_O_4_-graphene	~99	0.0097	[63]
WO_3_/g-C_3_N_4_	95	0.01897	[64]
Fe_2_TiO_5_	~100	0.016	[65]
T-BVO-600	98.93	0.0184	[66]

## Data Availability

All the data are available within the manuscript.
